# Frequency-Domain Second-Order Decorrelation with Compact Time-Domain Regularization for Convolutive Underwater Acoustic Source Separation

**DOI:** 10.3390/s26134189

**Published:** 2026-07-02

**Authors:** Huapeng Cao, Tingting Yang, Qi He, Ka-Fai Cedric Yiu

**Affiliations:** 1 School of Navigation, Dalian Maritime University, Dalian 116026, China; caohuapeng0001@dlmu.edu.cn (H.C.); yangtt@pcl.ac.cn (T.Y.); 2 Peng Cheng Laboratory, No. 2, Xingke 1st Street, Nanshan District, Shenzhen 518066, China; 3 Department of Applied Mathematics, The Hong Kong Polytechnic University, Hung Hom, Kowloon, Hong Kong, China; qivicky.he@connect.polyu.hk

**Keywords:** blind source separation, underwater acoustic propagation, joint diagonalization, time-domain constraint, ShipsEarBSS benchmark

## Abstract

Long-delay multipath pushes underwater acoustic mixing beyond the instantaneous model assumed by many classical algorithms; spectral overlap among mechanically and biologically generated sources compounds the difficulty, and low signal-to-noise ratios erode the higher-order statistical cues used by methods such as FastICA and JADE. This work adapts frequency-domain second-order decorrelation (FSD) to convolutive underwater mixtures by using multi-block joint diagonalization of cross-power spectral density matrices in the short-time Fourier transform domain together with compact time-domain regularization of the demixing filters. To provide a controlled and traceable evaluation, we introduce ShipsEarBSS, a simulated benchmark that combines single-source ShipsEar recordings with deep-water BELLHOP arrival responses to form virtual multichannel mixtures with known reference sources. Under a five-trial, eight-SNR protocol spanning −5 to 30 dB, an optimized compact FSD configuration is evaluated against the frozen reference FSD, PCA-SVD, and AuxIVA, and its main design choices are further examined through filter-length, multi-block CPSD, and output-ordering ablations. The results support a cautious conclusion: under the tested ShipsEarBSS protocol, compact time-domain regularization improves the FSD operating point, while the choices of filter support, CPSD block count, and output ordering remain empirical configuration decisions rather than universal optima.

## 1. Introduction

Maritime traffic and offshore resource exploitation have crowded the underwater acoustic environment. In an ocean waveguide, sound does not travel along a single path; refraction caused by sound-speed gradients, surface and bottom reflections, and scattering from internal turbulence generate multiple delayed and attenuated replicas at the receiver. A hydrophone therefore records a superposition of filtered source copies rather than a simple instantaneous mixture. Ambient noise varies in both space and time, with shipping noise concentrated mainly below 500 Hz and wind-driven surface agitation contributing broadband energy above 1 kHz. The resulting observations inherit long channel memory, strong spectral overlap, and often poor signal-to-noise ratios. Recovering source signals under these conditions demands blind source separation that can handle convolutive mixing in a non-stationary underwater channel with consistent fidelity.

Blind source separation seeks to recover source signals from observed mixtures without prior knowledge of either the sources or the propagation channels. Its classical development in speech separation offers useful intuition, but underwater acoustics imposes a harsher setting, with stronger multipath and much longer channel memory than most room-acoustic examples. BSS has been applied in mechanical fault diagnosis [1], EEG artifact removal [2], wireless communications [3], and biomedical imaging [4]. In the present setting, the signals recorded by hydrophones combine surface-ship noise, merchant-vessel noise, marine-animal vocalizations, submarine noise, and ambient ocean noise after transmission through an unknown underwater channel. These sources arise from distinct physical mechanisms and typically retain substantial statistical independence, which preserves the basic condition required for BSS. Practical success, however, has remained uneven. Kang [5], for example, developed a frequency-domain BSS-MVDR cascaded scheme for hydrophone arrays that improved bearing-estimation accuracy yet left the cross-frequency permutation ambiguity unresolved.

Modern sonobuoys and underwater sensor arrays routinely capture heavily mixed acoustic signatures from multiple vessels, marine mammals, and ambient sources. FastICA [6] and JADE [7] rely on higher-order statistics to enforce source independence, and that strategy weakens once convolution replaces instantaneous mixing. Each observation then contains delayed copies of every source, so the i.i.d. structure required by higher-order statistics no longer holds in any simple form. Low SNR makes the situation worse: fourth-order cumulants become difficult to estimate with useful precision, and separation quality deteriorates accordingly. SOBI [8] shifts attention to second-order information by jointly diagonalizing covariance matrices at multiple time lags. That choice better tolerates Gaussian noise, yet it still depends on assumptions that are awkward in underwater propagation, including accurate channel-length specification and a mixing process that remains sufficiently stationary over the analysis interval [9].

Recent underwater studies have explored several directions beyond classical ICA and SOBI. A multi-stage method that combines non-negative matrix factorization with minimum-mean-square-error denoising improved both reconstruction error and SDR under controlled conditions [10]. Gaeta [11] examined BSS for nonlinearly mixed shallow-water array data, while Wu et al. [12] coupled tensor decomposition with independent low-rank matrix analysis to exploit higher-order spatial structure and obtain a favorable initialization. Other developments include tensor-based formulations [13] and deep learning approaches [14], but these methods typically demand either larger training resources, stronger array assumptions, or heavier computation than is convenient in many practical deployment scenarios.

That tension appears clearly in recent application-specific designs. He et al. [15] cast underwater acoustic separation as a deep time-domain learning problem and used parallel dilated convolutions with grouped convolutions to enlarge the receptive field while limiting parameter growth. Yang et al. [16] worked in the spatial fractional Fourier domain and recovered rigid-scattering components through approximate joint diagonalization of generalized correlation matrices. Yuan [17] and Ganti [18] pursued direction-of-arrival estimation by combining blind separation with subsequent angular inference from recovered spatial signatures. Useful precedents, certainly. Yet none of them remove the need for a controlled, traceable evaluation chain tailored to convolutive underwater BSS under moderate SNR.

This paper focuses on that narrower problem: separating convolutively mixed underwater acoustic sources in the 0–20 dB regime without assuming precise array calibration or accepting excessive computational cost. The target is not a universal BSS solution. It is a method whose assumptions match the operating conditions of the benchmark and whose behavior can be audited end to end.

We therefore develop frequency-domain second-order decorrelation (FSD), an underwater BSS method centered on second-order statistics. Prior work has already considered cascades of second-order and higher-order criteria [19]; the distinguishing feature here is the adaptation and audited evaluation of the FSD framework for underwater convolutive mixtures. FSD performs multi-block cross-power spectral density (CPSD) joint diagonalization directly in the short-time Fourier transform (STFT) domain, so that the same separating system must decorrelate multiple temporal segments of a non-stationary mixture. The core demixing stage remains second-order. Complex-domain kurtosis ranking is treated only as an optional raw-output-ordering mechanism, while a compact time-domain filter-support constraint regularizes the separating filters and suppresses noise-driven overfitting. The contribution is therefore practical: this paper defines the traceable ShipsEarBSS benchmark, evaluates a compact FSD configuration against the frozen reference FSD and selected baselines, and reports ablations for the filter support, CPSD block count, and output-ordering step. These parameter choices are not claimed as independent methodological contributions or universal optima.

To evaluate FSD under conditions that remain physically interpretable and experimentally traceable, we constructed the ShipsEarBSS benchmark. ShipsEar supplies the single-source recordings. BELLHOP supplies the deep-water arrival responses [20]. Their combination yields multichannel convolutive mixtures with known reference sources and reproducible source-to-sensor propagation assignments, rather than ad hoc instantaneous sums or uncontrolled field recordings.

The remainder of this paper proceeds as follows: Section 2 formulates the convolutive mixing model and states the core assumptions. Section 3 describes the construction of ShipsEarBSS using BELLHOP-generated propagation templates. Section 4 presents the FSD algorithm, including the cost function, optimization procedure, compact time-domain regularization, and optional output-ordering heuristic. Section 5 reports qualitative and repeated-trial quantitative results across SNR conditions. Section 6 closes with the engineering boundaries of the present study and directions for future work.

## 2. Problem Formulation and System Model

This section states the underwater BSS problem in the form used throughout the paper. The presentation moves from the physical scene to the convolutive mixing model and then to the assumptions that make the separation problem identifiable and computationally tractable.

### 2.1. Scenario Description

**Figure 1:**
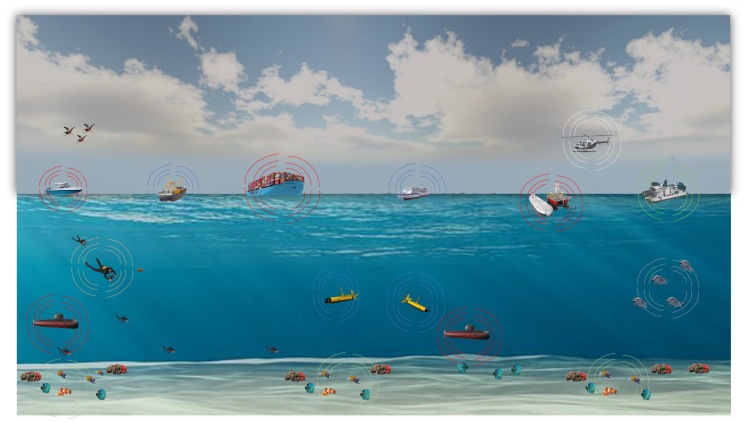
Schematic of the underwater acoustic BSS scenario. Multiple independent sources emit signals that propagate via multipath channels (direct path, surface/bottom reflections) to a hydrophone array, resulting in convolutive mixtures at each receiver.

Figure 1 depicts the target operating scene, in which different colored circles represent different types of acoustic sources. Multiple acoustic emitters may coexist in the same water column: surface vessels radiate hull and propeller noise, offshore platforms contribute low-frequency tonals, and marine mammals add bioacoustic components with their own temporal structure. Surface wave action, precipitation, and distant traffic supply a fluctuating ambient background. Their spectra overlap, sometimes strongly. At the receiver, direct arrivals mix with surface and bottom reflections, so the observed field depends not only on source content but also on propagation geometry and boundary interactions. The problem is therefore one of source separation in a multipath-dominated sound field rather than classification of isolated recordings.

The intended application is deep-water, medium-range passive acoustic monitoring, such as offshore surveillance-style source separation where multiple distant emitters may be observed by a fixed or slowly varying receiver configuration. The 1–11 km source–receiver range used in the BELLHOP arrival library is therefore a controlled set of candidate propagation distances for this medium-range deep-water benchmark. It is not meant to cover all underwater applications. Harbor monitoring, near-field operation, shallow-water channels, and rapidly time-varying scenes impose different propagation and array-calibration requirements and remain outside the validated scope of the present experiments.

Consider *M* statistically independent underwater sources, such as vessels, submerged vehicles, or marine mammals, observed by an array of *N* hydrophones. Propagation through the ocean introduces attenuation, delay spread, and frequency-dependent filtering before the signals reach the receivers. Each hydrophone therefore captures a superposition of filtered source contributions plus ambient noise. The task is to estimate the original source waveforms ${\left\{{\hat{s}}_{i}\left(t\right)\right\}}_{i=1}^{M}$ from the multichannel observations ${\left\{x_{j}\left(t\right)\right\}}_{j=1}^{N}$ alone, without access to detailed channel responses or source templates.

### 2.2. Convolutive Mixing Model

We model the propagation path from source *i* to hydrophone *j* as a linear time-invariant filter with finite impulse response $h_{ji}\left[\tau \right]$. Its length *L* captures the effective multipath delay spread retained in the model.

In the discrete-time domain, the signal observed at the *j*-th hydrophone is given by the convolutive mixture

(1) $$x_{j}\left[n\right]=\sum_{i=1}^{M}\sum_{\tau =0}^{L-1}h_{ji}\left[\tau \right]\;s_{i}\left[n-\tau \right]+v_{j}\left[n\right],\;j=1,\ldots ,N,$$

Here $s_{i}\left[n\right]$ denotes the *i*-th source signal, $x_{j}\left[n\right]$ the observation at hydrophone *j*, $h_{ji}\left[\tau \right]$ the τ-th channel tap from source *i* to hydrophone *j*, and $v_{j}\left[n\right]$ additive ambient noise, assumed to have zero mean and to be uncorrelated with the sources.

Vector notation makes the structure clearer. By defining the source vector as $s\left[n\right]={\left[s_{1}\left[n\right],\ldots ,s_{M}\left[n\right]\right]}^{T}$, the observation vector as $x\left[n\right]={\left[x_{1}\left[n\right],\ldots ,x_{N}\left[n\right]\right]}^{T}$, and the noise vector as $v\left[n\right]={\left[v_{1}\left[n\right],\ldots ,v_{N}\left[n\right]\right]}^{T}$, we obtain

(2) $$x\left[n\right]=\sum_{\tau =0}^{L-1}H\left[\tau \right]\;s\left[n-\tau \right]+v\left[n\right],$$

where H[τ] is the N×M mixing matrix whose (j,i)-th entry equals $h_{ji}\left[\tau \right]$. This is the standard MIMO convolutive mixing model.

FSD operates in the STFT domain, so we recast the model accordingly. When the analysis frame is sufficiently long relative to *L*, the time-domain convolution admits the usual narrowband approximation and becomes, at each frequency bin *f* and time frame *t*,

(3) X(f,t)=H(f)S(f,t)+V(f,t).

In this expression, X(f,t), S(f,t), and V(f,t) denote the STFT coefficients of the observations, sources, and noise. The matrix H(f) is the frequency-domain transfer matrix obtained from the DFT of ${\left\{H\left[\tau \right]\right\}}_{\tau =0}^{L-1}$.

The formulation rests on a few explicit assumptions. We focus on the determined setting, so the number of statistically independent sources is known a priori and satisfies M≤N, with the main experiments restricted to M=N. In an overdetermined case with more sensors than sources, whitening or dimensionality reduction could first project the observations onto an *M*-dimensional determined subspace before applying the same FSD demixing step; this extension is not experimentally evaluated here. The source signals ${\left\{s_{i}\left[n\right]\right\}}_{i=1}^{M}$ are assumed mutually independent, with vanishing crosscorrelation and higher-order cross-cumulants; at most one source may be Gaussian. In the underwater setting, AWGN and parts of the ambient background are often approximated as Gaussian perturbations, whereas ship-radiated machinery noise, cavitation, and marine-life vocalizations commonly retain non-Gaussian temporal and spectral structure. That condition matters chiefly because higher-order methods require non-Gaussianity to remove the rotational ambiguity left by second-order information alone. Within each short observation interval, the mixing filters $\left\{h_{ji}\left[\tau \right]\right\}$ are treated as LTI systems with finite support *L*. Real underwater channels do vary because of surface motion and internal waves, but the present study adopts a quasi-static approximation in which the channel coherence time exceeds the frame duration. The aim is modest and deliberate: isolate the core separation mechanism under controlled conditions before turning to genuinely time-varying propagation. The extension to time-varying scenarios via adaptive block-wise processing or recursive channel tracking is discussed as future work in Section 6.

### 2.3. Objective of Blind Source Separation

Under the model in (2), or equivalently (3) after STFT approximation, the objective is to estimate either a set of *separation filters* {W[ζ]} or a frequency-domain separation matrix W(f) such that

(4) $$y\left[n\right]=\sum_{\zeta }W\left[\zeta \right]\;x\left[n-\zeta \right]\;\mathrm{or}\;Y\left(f,t\right)=W\left(f\right)\;X\left(f,t\right)$$

approximates the original source vector s[n] up to the standard ambiguities of blind separation. In the present convolutive setting, these ambiguities include arbitrary scaling, a constant permutation of the source order, and a possible frequency-dependent phase term.

Section 3 next turns to benchmark construction, after which Section 4 introduces the FSD algorithm used to estimate W(f) by jointly diagonalizing second-order statistics across multiple time blocks, with optional raw output ordering and a common evaluation-stage gain alignment for scale-sensitive metrics.

## 3. ShipsEarBSS Benchmark Construction

This section defines how ShipsEarBSS is constructed for the BSS experiments. The emphasis falls on the provenance of the source material, the BELLHOP-derived propagation templates, and the rules used to assemble traceable multichannel mixtures.

### 3.1. Source Material and Propagation

To evaluate BSS algorithms under controlled underwater multipath conditions, we constructed a reproducible benchmark, denoted ShipsEarBSS, from the publicly available ShipsEar database [21]. ShipsEar enters this study only as a library of single-source underwater recordings. The multichannel convolutive mixtures are synthesized separately by assigning deep-water BELLHOP arrival responses to the source–sensor paths. ShipsEarBSS should therefore be read as a simulated BSS benchmark built from real source recordings and modeled propagation templates, not as a measured multihydrophone sea-trial dataset.

The ShipsEar database contains ship-radiated noise recordings collected in coastal environments and spanning 11 vessel types at a sampling rate of 52,734 Hz. We retained the five categories listed in Table 1. The choice was guided by criteria directly relevant to BSS evaluation: coverage of the 10 Hz–10 kHz band, variation in non-Gaussian structure, and clear differences in temporal modulation. Fishing vessels, for instance, tend to produce impulsive components, whereas ocean liners approach a more Gaussian character; dredgers show rhythmic pulsing, whereas passenger ferries radiate a more continuous broadband signature. The resulting set forces the separation algorithms to handle both spectral overlap and statistical diversity.

Each ShipsEar recording then passed through the same preprocessing pipeline. We segmented the signals into non-overlapping 5 s clips, downsampled them to 16 kHz, centered them to zero mean, and normalized them to unit variance. The downsampling step reduces computational cost while preserving the dominant low-frequency content that characterizes much of ship-radiated noise. After preprocessing, the clips are treated as single-source source material $s_{i}\left[n\right]$ for BSS mixture construction rather than as observations to be separated directly.

### 3.2. Underwater Acoustic Channel Simulation with BELLHOP

To generate the convolutive mixing paths, we used the BELLHOP ray-tracing model in a deep-water propagation scenario. The audited environment file specifies a water depth of approximately 3462 m, an explicit depth-dependent sound-speed profile, one receiver depth at 10 m, six candidate source depths spanning 100–1100 m, and six candidate ranges spanning 1–11 km. The corresponding arrivals serve as propagation templates from which the source-to-hydrophone responses are assembled:

- Environment: A deep-water environment with a water depth of approximately 3462 m was modeled. The sound-speed profile was depth-dependent and was specified explicitly in the BELLHOP environment file used for channel generation.
- Seabed: The bottom boundary parameters were specified in the BELLHOP environment file and kept fixed for all generated channels.
- Source–Receiver Geometry: The propagation template uses a receiver depth of 10 m, six candidate source depths from 100 m to 1100 m, and six source ranges from 1 km to 11 km, yielding 36 source–position combinations. For the BSS benchmark, one arrival response is assigned to each source–hydrophone pair by selecting from this deep-water arrival set. The current benchmark therefore represents a virtual five-sensor source-to-sensor assignment rather than a confirmed physical uniform linear array.

For each selected source–position pair (r,z), BELLHOP returns the complex amplitudes $A^{\left(r,z\right)}=\left[A_{1},A_{2},\ldots ,A_{P}\right]$ and absolute travel times $\tau^{\left(r,z\right)}=\left[\tau_{1},\tau_{2},\ldots ,\tau_{P}\right]$ of the *P* valid ray paths. In the implemented benchmark, the arrivals are applied in the frequency domain, while the associated amplitudes and delays are retained as metadata. An equivalent discrete-time CIR representation is

(5) $$h^{\left(r,z\right)}\left[m\right]=\sum_{p=1}^{P}{\tilde{A}}_{p}\cdot \delta \left[m-\left\lfloor \frac{\Delta \tau_{p}}{T_{s}}\right\rfloor \right],$$

where ${\tilde{A}}_{p}=A_{p}/\max(|A_{p}\left|\right)$ is the normalized complex amplitude, $\Delta \tau_{p}=\tau_{p}-\min\left(\tau \right)$ is the delay relative to the first arrival, $T_{s}=1/f_{s}$ is the sampling period, and ⌊·⌋ denotes the floor operation.

ShipsEarBSS is assembled in two stages. Each preprocessed ShipsEar clip is first paired with a source-to-hydrophone arrival response. The propagated contribution for one source–sensor path is written as

(6) $$y^{\left(r,z\right)}\left[n\right]=F^{-1}\left\{F\left\{s_{i}\left[n\right]\right\}\cdot F\left\{h^{\left(r,z\right)}\left[m\right]\right\}\right\},$$

where F and $F^{-1}$ denote the FFT and inverse FFT. The mixture is then formed by assigning independent source clips ${\left\{s_{i}\left[n\right]\right\}}_{i=1}^{M}$ to source-to-hydrophone responses $\left\{h_{ji}\left[n\right]\right\}$, propagating them individually, and summing the contributions at each hydrophone:

(7) $$x_{j}\left[n\right]=\sum_{i=1}^{M}\left(s_{i}*h_{ji}\right)\left[n\right]+v_{j}\left[n\right],\;j=1,\ldots ,N,$$

where $v_{j}\left[n\right]$ denotes additive white Gaussian noise (AWGN) added after the clean mixtures have been assembled, so that the observation SNR can be controlled explicitly. This separation of roles matters. ShipsEar supplies realistic source material; BELLHOP supplies reproducible deep-water propagation templates; the final observations are multichannel convolutive mixtures rather than single-channel classification samples. When Ocean Waves are included as one of the five source classes, they are treated as structured reference sources to be separated, not as interchangeable with the AWGN perturbation $v_{j}\left[n\right]$. The complete construction procedure is summarized in Algorithm 1.
**Algorithm 1** Construction of the ShipsEarBSS Benchmark for BSS.**Require:** ShipsEar single-source audio clips, BELLHOP arrival files for deep-water source positions.**Ensure:** ShipsEarBSS benchmark with reference sources, channel contributions, clean mixtures, noisy mixtures, and metadata. 1:**Preprocess Sources:** Segment, downsample, center, and normalize ShipsEar files.2:**Define Trial Manifest:** For each trial seed, select one source clip from each source class.3:**Assign Channels:** For every source–sensor pair, select one BELLHOP arrival response from the 6-by-6 range/depth library and record the corresponding range/depth indices.4:**Apply Propagation:** Filter each selected source by its assigned arrival response using Equation (6).5:**Assemble Clean Mixtures:** Sum the propagated source contributions at each virtual sensor using Equation (7) with $v_{j}\left[n\right]=0$.6:**Generate Noisy Mixtures:** Add AWGN after clean mixture construction for each target SNR.7:**Store Evidence:** Save reference sources, clean mixtures, noisy observations, source–sensor contributions, arrival amplitudes/delays, trial seeds, and channel-assignment metadata.

## 4. Frequency-Domain Decorrelation-Based Hybrid Blind Source Separation Algorithm

This section develops the complete FSD algorithm. The emphasis is on the signal-processing rationale behind each design choice: why the demixing stage is posed in the STFT domain, why multi-block CPSD matrices are jointly diagonalized, and why the separating filters are regularized in the time domain after each frequency-domain update. The target setting remains the same throughout: determined underwater array observations corrupted by long-delay multipath, overlapping source spectra, and additive noise over the −5 to 30 dB range.

### 4.1. Algorithm Overview

**Figure 2:**
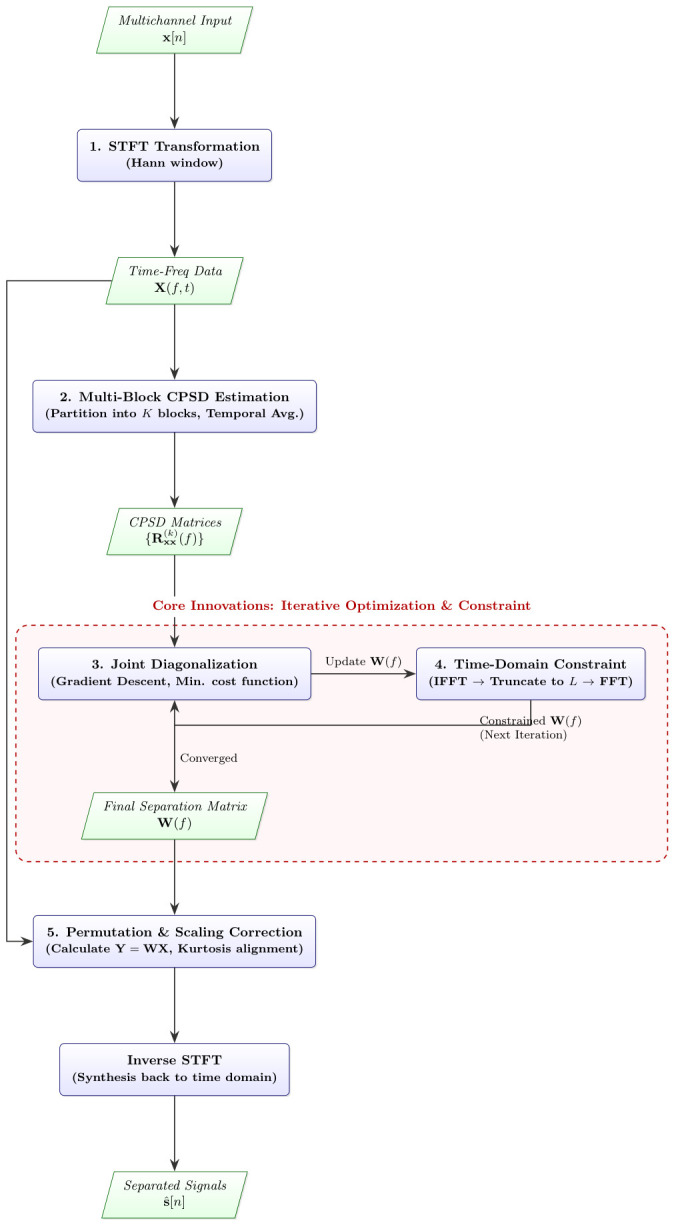
Block diagram of the FSD algorithm. The core innovations, namely, multi-block CPSD joint diagonalization and the time-domain filter-length constraint, are highlighted.

FSD operates in the STFT domain, where a long convolutive mixture can be approximated as a collection of narrowband instantaneous mixtures. For underwater array data, that approximation is useful but not sufficient on its own: the demixing matrix must still be constrained so that the reconstructed filters remain physically plausible in the time domain. Figure 2 summarizes the processing chain.

1. STFT Transformation: The multichannel observation $x\left[n\right]\in R^{N\times T}$ is mapped to the time–frequency plane with a Hamming-window STFT using FFT size $N_{\mathrm{FFT}}$ and a hop size of $N_{\mathrm{FFT}}/4$, corresponding to 75% overlap. The audited configurations report the specific FFT size used in each experiment. This produces complex coefficients $X\left(f,t\right)\in C^{N\times F\times T_{f}}$.
2. Multi-Block Cross-Power Spectral Density (CPSD) Estimation: The time frames are partitioned into *K* blocks, and each block yields a CPSD matrix $R_{\mathrm{xx}}^{\left(k\right)}\left(f\right)$ at every frequency bin.
3. Joint Diagonalization via Gradient Descent: A separation matrix $W\left(f\right)\in C^{N\times N}$ is initialized as the identity and iteratively updated so that the transformed CPSD matrices lose as much off-diagonal energy as possible across all *K* blocks.
4. Time-Domain Filter-Length Constraint: After each frequency-domain update, W(f) is mapped back to the time domain, truncated to length *L*, and transformed forward again. This step suppresses unrealistically long demixing responses and stabilizes the optimization in noise-dominated bands.
5. Optional Output Ordering: The separated components Y(f,t)=W(f)X(f,t) may undergo heuristic raw output ordering, for instance, through complex-domain kurtosis ranking before iSTFT. Scale ambiguity is handled consistently during metric computation through gain alignment and is not treated here as an independent algorithmic contribution.

Algorithm 2 states the full procedure. The discussion below focuses on the two ingredients that matter the most in the present underwater setting: the use of multi-block second-order statistics to exploit source non-stationarity, and the time-domain regularization that keeps the demixing filters compact even when the propagation channel itself is long.
**Algorithm 2** The FSD algorithm.**Require:** Multichannel observation signal $x\left[n\right]\in R^{N\times T}$, Hamming-window STFT with FFT size $N_{\mathrm{FFT}}$ and hop size $N_{\mathrm{FFT}}/4$ (75% overlap), number of CPSD blocks *K*, compact demixing-filter support *L*, learning rate η=0.5, max iterations max_iter, convergence threshold ϵ.**Ensure:** Estimated source signals $\hat{s}\left[n\right]\in R^{N\times T}$.  1:**Preprocessing:** Center x[n] and optionally whiten.  2:**STFT:** Compute X(f,t) using a Hamming window, FFT size $N_{\mathrm{FFT}}$, and hop size $N_{\mathrm{FFT}}/4$.  3:Let *F* = number of frequency bins, $T_{f}$ = number of time frames.  4:Partition frames into *K* blocks, compute CPSD $R_{\mathrm{xx}}^{\left(k\right)}\left(f\right)$ via Equation (8).  5:Compute normalization factors m(f) via Equation (11).  6:Initialize $W\left(f\right)\leftarrow I_{N}$ for all *f*.  7:**for** f=1 to *F* **do**  8:  **for** iter=1 to max_iter **do**  9:    $E^{\left(k\right)}\left(f\right)\leftarrow W\left(f\right)R_{\mathrm{xx}}^{\left(k\right)}\left(f\right)W^{H}\left(f\right)$, ∀k.10:    Compute J(W(f)) via Equation (10).11:    **if** iter>1 and $|J-J_{\mathrm{old}}|<\epsilon$ **then**12:        **break**13:    **end if**14:    $J_{\mathrm{old}}\leftarrow J$.15:    Compute $E_{\mathrm{off}}^{\left(k\right)}\left(f\right)=E^{\left(k\right)}\left(f\right)-\mathrm{diag}\left(E^{\left(k\right)}\left(f\right)\right)$.16:    Compute gradient G(f) via Equation (14).17:    Remove diagonal: $G_{\mathrm{corr}}\left(f\right)=G\left(f\right)-\mathrm{diag}\left(G\left(f\right)\right)$.18:    Update: $W\left(f\right)\leftarrow W\left(f\right)-\eta G_{\mathrm{corr}}\left(f\right)$.19:    **Time-domain constraint:**20:        $w_{t}\left[\tau \right]\leftarrow F^{-1}\left\{W\left(f\right)\right\}$ (IFFT along frequency).21:        Truncate $w_{t}\left[\tau \right]$ to length *L* (set τ≥L to zero).22:        $W\left(f\right)\leftarrow F\{w_{t}\left[\tau \right]\}$ (FFT back).23:  **end for**24:**end for**25:**Separate:** Y(f,t)=W(f)X(f,t).26:**Optional output ordering:** If enabled, compute kurtosis $\kappa_{i}\left(f\right)$ via Equation (18) and rank the separated STFT output channels before iSTFT to standardize the raw output order.27:**Synthesis:** $\hat{s}\left[n\right]\leftarrow \mathrm{iSTFT}\left(Y\left(f,t\right)\right)$.28:**Evaluation note:** Scale-sensitive metrics are computed after common gain alignment; this is not an FSD demixing step.

### 4.2. Multi-Block CPSD Joint Diagonalization

Conventional second-order BSS methods typically diagonalize either one covariance matrix or a small set of fixed time-lagged covariances. FSD instead works with multiple CPSD matrices [22] extracted from successive temporal segments of the same recording. That distinction is important in underwater acoustics. Ship-radiated noise is rarely stationary over the full observation interval: propeller loading changes with motion, machinery signatures pulse, and bioacoustic emissions appear intermittently. The spatial covariance structure therefore drifts from block to block even when the propagation geometry remains approximately fixed. Joint diagonalization across those blocks turns that drift into a separation cue.

Let $X\left(f,t\right)\in C^{N}$ denote the STFT coefficient vector at frequency bin *f* and time frame *t*. The STFT was implemented with a Hamming window and a hop size of $N_{\mathrm{FFT}}/4$, corresponding to 75% overlap. The FFT size $N_{\mathrm{FFT}}$ is configuration-specific and is reported with the corresponding audited experiment. The total number of frames is denoted by $T_{f}$.

To capture temporal variations, the frames are divided into *K* consecutive blocks. The block count controls a performance–complexity trade-off: smaller *K* gives longer blocks and lower per-block estimation variance, whereas larger *K* captures finer non-stationarity but increases runtime and may increase the variance of each local CPSD estimate. The audited compact configuration uses K=5 as a practical compromise, not as a universal optimum; the sensitivity to *K* is reported in Section 5.4. For the *k*-th block (k=0,…,K−1), the CPSD matrix at frequency *f* is estimated as

(8) $$R_{\mathrm{xx}}^{\left(k\right)}\left(f\right)=\frac{1}{T_{\mathrm{block}}}\sum_{t=t_{k}}^{t_{k}+T_{\mathrm{block}}-1}X\left(f,t\right)X^{H}\left(f,t\right),$$

where $T_{\mathrm{block}}=\left\lfloor T_{f}/K\right\rfloor$, $t_{k}=k\cdot T_{\mathrm{block}}$, and ${\left(\cdot \right)}^{H}$ denotes the Hermitian transpose. Each frequency bin therefore carries *K* CPSD matrices, each tied to a different portion of the observation window and hence to a different local source-activity pattern.

The separation matrix $W\left(f\right)\in C^{N\times N}$ is applied to the observed coefficients to obtain the separated components:

(9) Y(f,t)=W(f)X(f,t).

Ideally, the output CPSD matrices $R_{\mathrm{yy}}^{\left(k\right)}\left(f\right)=W\left(f\right)R_{\mathrm{xx}}^{\left(k\right)}\left(f\right)W^{H}\left(f\right)$ become diagonal for every block *k*. In that case, the separated components are mutually uncorrelated within each local time segment, which is precisely the second-order condition FSD tries to enforce.

We quantify the degree of diagonalization by the sum of squared off-diagonal elements across all blocks. The cost function for frequency *f* is defined as:

(10) $$J\left(W(f)\right)=\sum_{k=0}^{K-1}\sum_{i\neq j}{\left|{\left[W\left(f\right)R_{\mathrm{xx}}^{\left(k\right)}\left(f\right)W^{H}\left(f\right)\right]}_{ij}\right|}^{2}.$$

Minimizing J(W(f)) encourages W(f) to diagonalize all *K* CPSD matrices simultaneously. A single block supplies only one local second-order estimate. Several blocks, if their second-order structure differs enough, can sharpen the demixing problem, but the empirical effect depends on the tested protocol and the cost of estimating additional CPSD matrices.

Numerical conditioning varies strongly across frequency because underwater source spectra and channel transfer functions are highly uneven. We therefore assign each frequency bin a normalization factor m(f) that normalizes the aggregate CPSD power:

(11) $$m\left(f\right)=\frac{1}{\sum_{k=0}^{K-1}{∥R_{\mathrm{xx}}^{\left(k\right)}\left(f\right)∥}_{F}^{2}+\varepsilon },$$

where ${∥\cdot ∥}_{F}$ denotes the Frobenius norm and $\varepsilon =10^{-12}$ prevents division by zero. This factor enters the gradient update so that a few high-energy bands do not dominate the optimization.

The objective J(W(f)) is non-convex, so no useful closed-form solution is available. FSD therefore updates W(f) iteratively through a complex-valued gradient descent.

We define the transformed CPSD matrix for block *k* as $E^{\left(k\right)}\left(f\right)=W\left(f\right)R_{\mathrm{xx}}^{\left(k\right)}\left(f\right)W^{H}\left(f\right)$. The off-diagonal part of $E^{\left(k\right)}\left(f\right)$ is obtained by setting its diagonal entries to zero:

(12) $$E_{\mathrm{off}}^{\left(k\right)}\left(f\right)=E^{\left(k\right)}\left(f\right)-\mathrm{diag}\left(E^{\left(k\right)}\left(f\right)\right).$$

The gradient of J(W(f)) with respect to the complex matrix W(f) (treating W and its conjugate $W^{*}$ as independent variables) can be derived as:

(13) $$\nabla_{W^{*}}J=2\sum_{k=0}^{K-1}E_{\mathrm{off}}^{\left(k\right)}\left(f\right)\;W\left(f\right)R_{\mathrm{xx}}^{\left(k\right)}\left(f\right).$$

In practice, we compute the gradient matrix G(f) as:

(14) $$G\left(f\right)=2\;m\left(f\right)\sum_{k=0}^{K-1}E_{\mathrm{off}}^{\left(k\right)}\left(f\right)\;\left[W\left(f\right)R_{\mathrm{xx}}^{\left(k\right)}\left(f\right)\right].$$

A raw gradient step may perturb the diagonal terms of $E^{\left(k\right)}\left(f\right)$ even though those terms do not contribute to the cost. That perturbation adds numerical clutter without helping separation. We therefore remove the diagonal part of G(f) before updating:

(15) $$G_{\mathrm{corr}}\left(f\right)=G\left(f\right)-\mathrm{diag}\left(G(f)\right).$$

The separation matrix is then updated using a learning rate η:

(16) $$W_{\mathrm{new}}\left(f\right)=W_{\mathrm{old}}\left(f\right)-\eta \;G_{\mathrm{corr}}\left(f\right).$$

The frequency-domain demixing matrices alone do not guarantee physically sensible separating filters. To constrain their effective support, we map W(f) back to the time domain after each update. Let $w_{t}\left[\tau \right]\in R^{N\times N}$ denote the corresponding impulse response. We retain only the first *L* taps and set the remainder to zero:

(17) $$w_{t}\left[\tau \right]=\left\{\begin{array}{cc} w_{t}\left[\tau \right], & 0\leq \tau <L,\\ 0, & \tau \geq L. \end{array}\right.$$

The constrained matrix $W_{\mathrm{con}}\left(f\right)$ is then recomputed by the FFT. The parameter *L* is the compact support imposed on the demixing filters and is therefore a regularization parameter rather than the physical BELLHOP channel length. This distinction is important because the current ShipsEarBSS benchmark applies BELLHOP arrivals through arrival-based filtering and does not materialize a fixed-length channel impulse response for each path. At 16 kHz, the tested values L=64,128,256, and 512 correspond to approximately 4, 8, 16, and 32 ms, respectively. The ablation in Section 5.4 evaluates this sensitivity; it does not imply that *L* covers the full physical propagation memory or that one value is universally optimal.

The resulting optimization problem lives in a non-convex complex domain, and the time-domain truncation makes strict convergence analysis difficult. Still, the frequency-wise normalization by m(f) acts as an effective pre-conditioner, keeping the gradient scale under control across bands. We therefore use a fixed learning rate η=0.5 rather than an elaborate decay schedule. In the audited experiments reported below, iteration stops once the relative change in the cost falls below $\epsilon =10^{-6}$ or the update count reaches max_iter=5000. No numerical divergence or case-level failure was observed in the audited runs with this fixed setting, so it is retained as a reproducible engineering choice rather than a general convergence guarantee.

### 4.3. Optional Output Ordering and Scale Ambiguity Handling

The separation matrices W(f) produced by the gradient descent stage still inherit the familiar ordering and scaling ambiguities of frequency-domain BSS. In this manuscript, output ordering is treated as an optional algorithmic mechanism for standardizing the raw order of reconstructed outputs. Scale ambiguity is handled consistently in the evaluation stage through gain alignment before computing SDR and correlation; algorithmic scaling restoration is not treated as an independent contribution in this study.

Many underwater man-made sources are visibly non-Gaussian, whereas ambient background fluctuations often lie closer to Gaussian behavior. This contrast motivates the use of complex-domain kurtosis as one possible output-ordering heuristic. For a complex random variable *Y*, the normalized kurtosis is

(18) $$\kappa \left(Y\right)=\frac{{E[|Y|}^{4}]-2{\left(E[|Y{|}^{2}]\right)}^{2}-|E\left[Y^{2}\right]{|}^{2}}{{\left(E[|Y{|}^{2}]\right)}^{2}}.$$

For super-Gaussian sources, κ(Y)>0; for Gaussian noise, κ(Y)=0. Cavitation and rotating machinery often generate impulsive waveforms with heavy-tailed amplitude statistics, so large positive kurtosis values are physically plausible for several ship-radiated components. For each frequency bin *f* and output channel *i*, we compute $\kappa_{i}\left(f\right)$ from $Y_{i}\left(f,t\right)={\left[W\left(f\right)X\left(f,t\right)\right]}_{i}$. Sorting channels by descending $\kappa_{i}\left(f\right)$ before iSTFT changes the raw algorithmic output order. It should be interpreted as an output-ordering heuristic, not as a solution to the general frequency-domain permutation ambiguity. The evaluation-time reference assignment used for SDR, correlation, MSE and the SIR is a metric-computation step.

Because blind source separation has inherent scale ambiguity, separated components may have arbitrary gains. Rather than claiming an algorithmic scaling-restoration module, this study applies the same gain alignment to all evaluated methods before computing scale-sensitive metrics. This convention makes the reported SDR and correlation comparable across algorithms without treating scaling correction as a separate FSD contribution.

With the final W(f), the separated STFT coefficients Y(f,t) are computed first. If optional ordering postprocessing is enabled, the output channels of Y(f,t) are re-ordered before synthesis. An inverse STFT with the same Hamming-window and $N_{\mathrm{FFT}}/4$-hop settings then returns the time-domain source estimates $\hat{s}\left[n\right]\in R^{N\times T}$.

### 4.4. Methodological Rationale for Underwater Acoustic Scenarios

The design choices embedded in FSD address several features that recur in underwater array recordings and are often troublesome for generic BSS methods.

By operating in the frequency domain and jointly diagonalizing multiple CPSD matrices, FSD can address long multipath channels without explicit identification of the channel impulse responses themselves. That stands in contrast to time-domain ICA methods such as FastICA and JADE, whose instantaneous-mixing assumptions are poorly matched to delayed underwater propagation. It also differs from SOBI, whose time-lagged covariances become less discriminative when delay spread is large and source activity changes within the analysis window.

Second-order criteria also interact more favorably with additive Gaussian noise than higher-order ones, because Gaussian noise contributes primarily to the diagonal of the CPSD matrices and does not directly bias the off-diagonal terms that drive the objective. This point is especially relevant in low-SNR underwater recordings, where fourth-order statistics are often poorly estimated. The explicit time-domain truncation adds a second layer of protection by discouraging demixing filters from chasing narrow, noise-dominated spectral fluctuations.

Many underwater sources are strongly non-stationary over the time scale of a few STFT blocks: fishing boats show pulsed harmonics, drilling platforms generate rhythmic impacts, and whale calls appear intermittently. FSD exploits that fact directly. The same separation matrix must diagonalize second-order statistics extracted from different time intervals, so temporal variability becomes an ally rather than a nuisance.

When enabled, complex-domain kurtosis provides a lightweight ordering heuristic rooted in the non-Gaussian statistics of many man-made underwater sources. Unlike correlation-based schemes, it does not require reference signals or exhaustive pair-wise matching across bins. Its role here remains limited, however. The repeated-trial conclusions in Section 5.3 depend on the audited full-rerun configuration and should not be read as evidence that kurtosis ranking resolves cross-frequency ambiguity in general.

FSD does require iterative optimization, but the resulting cost remains manageable for the array sizes considered here (N≤10). The dominant per-iteration expense is the set of *K* matrix multiplications of size N×N, yielding the familiar $O(KN^{3})$ scaling. For fixed *K*, *N*, and STFT resolution, the method remains suitable for structured offline analysis and batch evaluation; the empirical runtime consequences of the audited configurations are reported in Section 5.3.

The next section evaluates these claims experimentally and then restricts the formal quantitative comparison to the traceable algorithmic baseline set retained in the revised evidence chain.

## 5. Results and Discussion

This section reports the experimental evaluation of FSD in MATLAB (R2025b) on a workstation equipped with an Intel Core i5-8300H CPU at 2.30 GHz and 8 GB RAM. The mixtures were generated from ShipsEarBSS source clips, after which additive Gaussian noise was introduced to emulate controlled observation conditions. Real deployment does not reveal the source count in advance, but a fixed determined setting remains the standard starting point for method validation because it permits like-for-like comparison under a reproducible protocol. The discussion below follows that convention.

### 5.1. Dataset and Simulation Setup

The experiments use the ShipsEarBSS benchmark described in Section 3.1. Single-source clips from the ShipsEar database provide the source material, while deep-water BELLHOP arrivals define the source-to-hydrophone propagation templates needed to assemble multichannel convolutive mixtures.

The propagation model follows the audited deep-water BELLHOP configuration summarized in Table 2. For each trial, every source–sensor pair receives one arrival response selected from the 6-by-6 range/depth library, and the multichannel observations are formed by summing the propagated source contributions at each virtual hydrophone.

The reported results are obtained in a determined benchmark with M=5 independent source clips and N=5 virtual hydrophones. The selected clips span distinct underwater acoustic signatures, and each source propagates toward each hydrophone through one assigned deep-water response. AWGN is added only after clean mixture construction so that the observation SNR is controlled independently of the convolutive channel synthesis.

The resulting scene contains five independent acoustic sources observed by five virtual hydrophones. The source set includes vessel-radiated sounds, whale vocalizations, and ocean-wave recordings (summarized in Table 3). In the benchmark, Ocean Waves remain structured reference sources to be separated, whereas AWGN enters later as an unstructured perturbation used only for SNR-controlled testing.

The received signal vector x(t) was modeled as the convolution of source signals with channel impulse responses:

(19) $$x\left(t\right)=\sum_{n=1}^{5}h_{n}\left(t\right)*s_{n}\left(t\right)+n\left(t\right),$$

where $s_{n}\left(t\right)$ denotes the *n*-th source recording, $h_{n}\left(t\right)$ denotes the associated vector of multipath channel responses drawn from the BELLHOP arrival library, and n(t) denotes AWGN.

**Figure 3:**
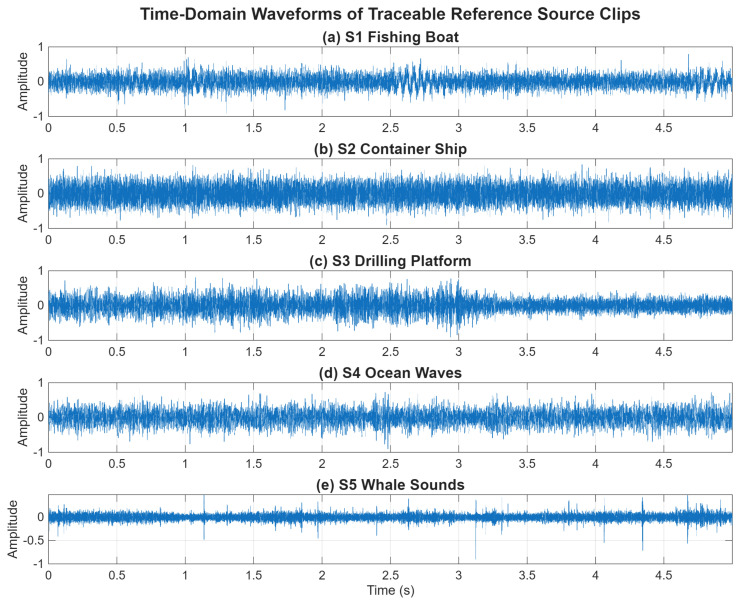
Time-domain waveforms of the five reference source clips in the representative ShipsEarBSS trial: (**a**) Fishing Boat, (**b**) Container Ship, (**c**) Drilling Platform, (**d**) Ocean Waves, and (**e**) Whale Sounds.

**Figure 4:**
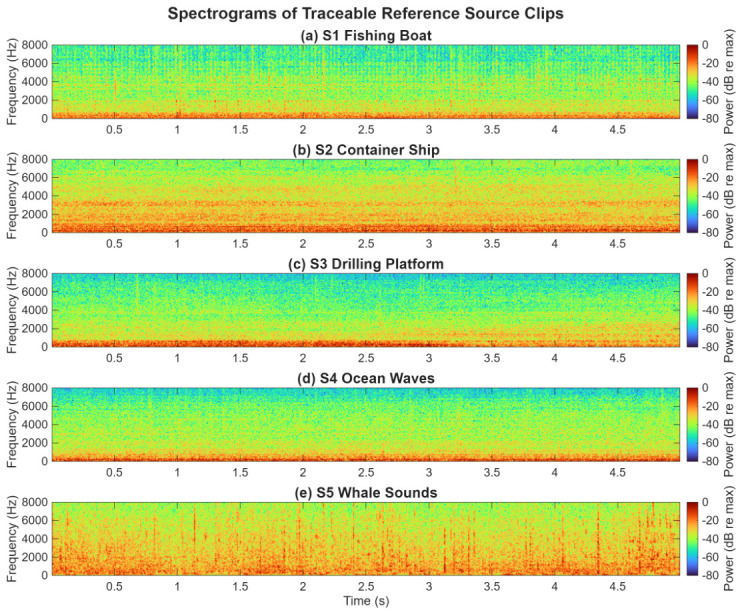
Spectrograms of the five reference source clips with a common dynamic range of −80 to 0 dB relative to the maximum power: (**a**) Fishing Boat, (**b**) Container Ship, (**c**) Drilling Platform, (**d**) Ocean Waves, and (**e**) Whale Sounds.

**Figure 5:**
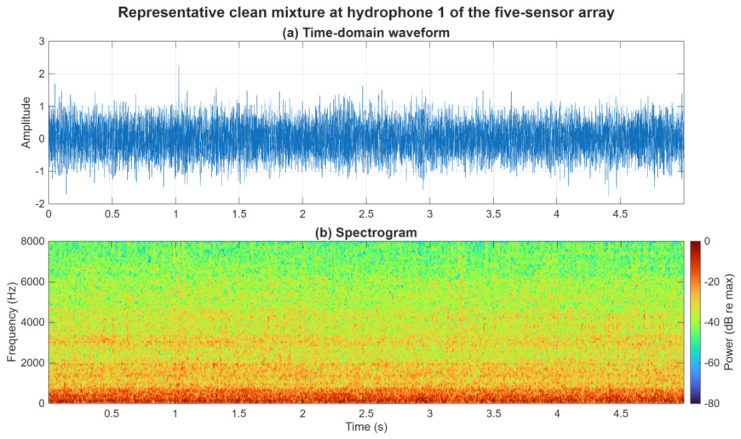
Representative clean convolutive mixture observed at hydrophone 1 of the virtual five-sensor array: (**a**) time-domain waveform and (**b**) spectrogram. The plotted channel is a visualization of one array observation; the BSS algorithms use the multichannel mixture.

**Figure 6:**
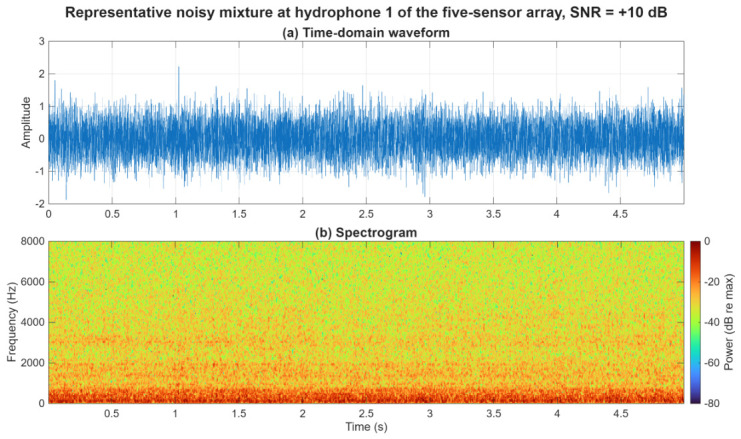
Representative noisy convolutive mixture observed at hydrophone 1 of the virtual five-sensor array after adding AWGN at 10 dB: (**a**) time-domain waveform and (**b**) spectrogram.

All signals were processed at 16 kHz. Across the FSD configurations, the STFT used a Hamming window and a hop size of one-quarter of the FFT size, corresponding to 75% overlap; the FFT size is reported for each audited configuration. The target SNR was swept from −5 to 30 dB. Figure 3, Figure 4, Figure 5 and Figure 6 illustrate one traceable trial used by the current processing chain. The figures are intended to document the source material and mixture construction; the separation claims are based on the repeated-trial quantitative results reported in Section 5.3.

### 5.2. Experimental Protocol and Statistical Reporting

In each BSS trial, five independent source clips were drawn from the ShipsEar-derived source library and propagated through deep-water BELLHOP responses assigned to the source–hydrophone pairs. The observations were then assembled according to Equation (7), and AWGN was added at the target SNR. Every algorithm was evaluated on the same mixtures at each SNR so that differences in performance could be attributed to the separation method rather than to a change in source realization or propagation path.

The current protocol uses five independent trials with seeds 1001–1005. Each trial contains five source clips, 25 source–sensor channel assignments, the clean multichannel mixtures, the reference sources, and metadata storing the selected range/depth indices. All quantitative metrics reported in Section 5.3 were computed only after the separated outputs had passed through the same evaluation-time alignment pipeline: delay compensation, amplitude alignment, and reference-source assignment. This common metric pipeline is essential because SDR and correlation would otherwise be confounded by trivial blind-separation ambiguities.

Figure 3 shows that the five reference clips differ substantially in temporal envelope and amplitude modulation, which is consistent with their different physical generation mechanisms. The corresponding spectrograms in Figure 4 make the separation problem more explicit. Several source classes occupy overlapping low-frequency regions before propagation, while their time–frequency structures differ in stationarity and bandwidth: the Drilling Platform clip contains comparatively steady low-frequency energy, the Ocean Waves clip forms a diffuse broadband background, and the Fishing Boat and Container Ship clips exhibit vessel-radiated low-frequency components with different temporal patterns. These partially overlapping but non-identical structures provide the statistical diversity exploited by the BSS algorithms after convolutive mixing.

After BELLHOP propagation, each hydrophone receives a different convolutive superposition of the five source clips. Figure 5 gives hydrophone 1 as a representative clean observation from the five-sensor array; the source-specific structures that were visible in Figure 4 are no longer separable by eye in a single received channel. Adding reproducible AWGN at the 10 dB operating point yields the noisy observation in Figure 6. This pair of figures documents the observation model, while the quantitative SNR sweep that follows evaluates performance over all tested noise levels and all five repeated trials.

### 5.3. Analysis of Separation Performance

All separation results reported here derive from the traceable ShipsEarBSS processing chain, which stores identical source clips, channel assignments, and additive-noise realizations for every evaluated SNR. Because each algorithm operates on exactly the same mixtures, the reported differences isolate the separation procedures rather than reflecting accidental variation in the simulated scene. Per-source metrics are evaluated on the reference-source side after delay compensation, gain alignment, and reference-source assignment. This convention removes the standard blind-separation ambiguities from the SDR and correlation values; the raw order of the separated outputs therefore carries no fixed physical-class meaning.

Within this framework, the optimized compact FSD denotes the selected compact configuration obtained under the audited full-rerun protocol. For the present study it uses $N_{\mathrm{FFT}}=256$ (code parameter T=256), K=5, L=64, time-domain truncation, and channel standardization. The component ablations in Section 5.4 instead fix $N_{\mathrm{FFT}}=512$ (code parameter T=512) so that the tested factor can be isolated under the reference FSD parameterization. The optimized compact FSD should be read as a parameterized FSD configuration, not as a new algorithmic family beyond FSD. It is compared against the frozen reference FSD, PCA-SVD, and AuxIVA under identical source assignments, noise realizations, and delay-compensated metric evaluation. The comparison therefore targets algorithmic BSS performance under the audited simulation protocol, not qualitative visual agreement in any single selected example.

Table 4 summarizes the traceable comparison set. Within this set, PCA-SVD reconstructs source estimates from the leading singular-vector projections of the noisy multichannel mixture, whereas AuxIVA serves as the frequency-domain convolutive BSS reference. At each tested SNR, the plotted mean SDR, correlation, and SIR values aggregate 25 matched-source metric rows, namely, five independent trials and five reference sources per trial after the common alignment pipeline of delay compensation, amplitude alignment.

**Figure 7:**
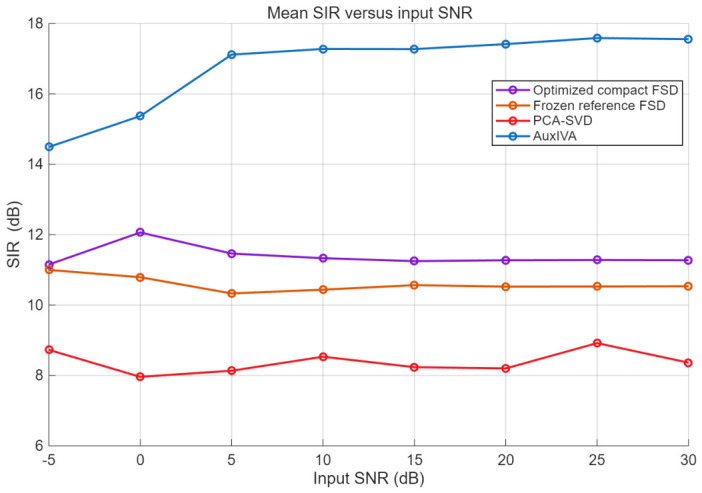
Mean SIR versus input SNR for the selected algorithmic baselines. Each point aggregates 25 matched-source metric rows at the corresponding SNR (5 trials × 5 reference sources).

Figure 7 adds the interference-related view requested for source-separation evaluation. The SIR is computed by the shared metric routine after delay, gain, and reference assignment. The optimized compact FSD improves the SIR relative to the frozen reference FSD and PCA-SVD overall.

**Figure 8:**
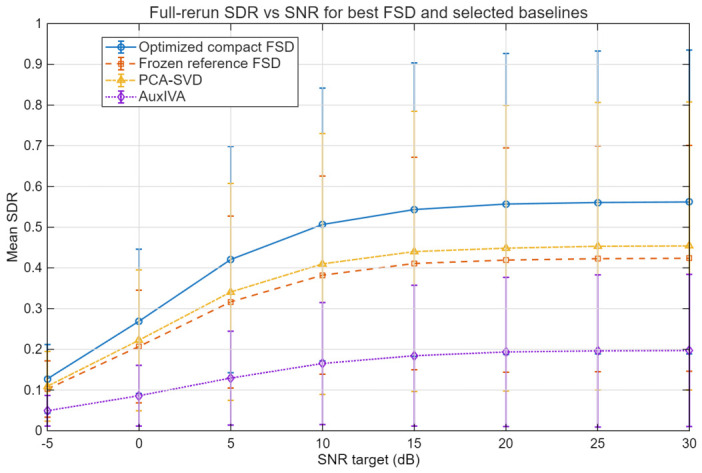
Mean SDR versus input SNR for the optimized compact FSD, frozen reference FSD, PCA-SVD, and AuxIVA. Error bars denote the standard deviation across the 25 matched-source metric rows available at each SNR under the unified ShipsEarBSS protocol. Positive upward separation of the optimized compact FSD curve indicates improved distortion suppression over the selected algorithmic baselines.

Figure 8 shows that the optimized compact FSD attains the highest mean SDR at every tested SNR from −5 to 30 dB within the selected algorithmic comparison group. The improvement over the frozen reference FSD is already positive at the lowest tested SNR and grows from approximately 0.025 dB at −5 dB to about 0.138 dB at 30 dB. The same monotone advantage appears relative to PCA-SVD and AuxIVA. To quantify the uncertainty of these small gains, we also computed paired SDR differences on the matched trial–SNR–source rows. The overall paired mean gain over the frozen reference FSD is 0.108 dB, with a 95% paired mean confidence interval of [0.083,0.132] dB; the corresponding intervals versus PCA-SVD and AuxIVA are [0.066,0.101] dB and [0.251,0.335] dB, respectively. The by-SNR paired intervals against the frozen reference FSD also remain positive in Table 5. These statistics support a reproducible benchmark-level advantage under the audited protocol, while the small absolute gains should not be interpreted as a guarantee of perceptible improvement in every practical deployment.

**Figure 9:**
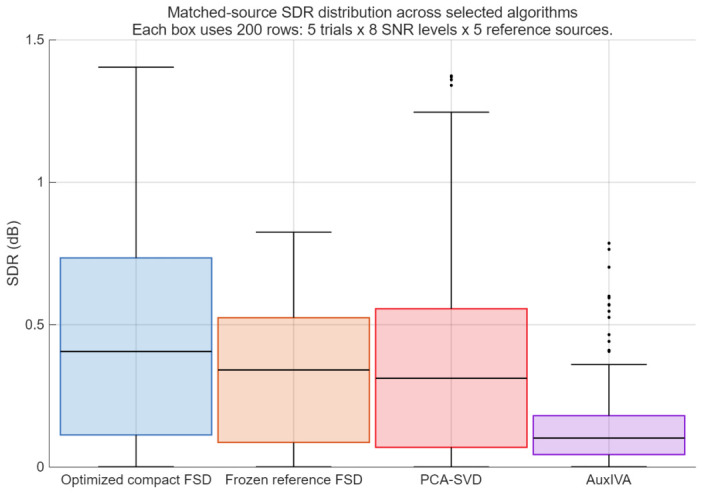
Box plot of matched-source SDR distributions for the selected algorithmic comparison group. Each box uses 200 matched-source SDR rows aggregated over five trials, eight SNR levels, and five reference sources after the common delay, gain, and reference-assignment pipeline. This distributional view complements the mean SDR curves and avoids relying only on average SDR performance.

The box plot in Figure 9 shows the spread of SDR values across trials, SNR levels, and reference sources. The optimized compact FSD has the highest overall mean SDR in the selected comparison group, but the distributions also show substantial overlap across methods and source/SNR conditions. The result should therefore be read as a benchmark-level empirical advantage under the audited protocol, not as a guarantee for every individual mixture.

**Figure 10:**
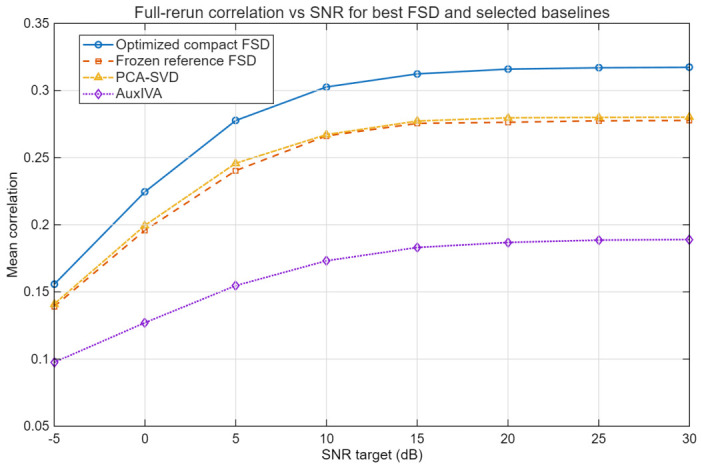
Mean waveform correlation versus input SNR for the selected algorithmic baselines. Each point aggregates the same 25 matched-source metric rows used in Figure 8 at the corresponding SNR (5 trials × 5 reference sources). Higher correlation indicates that the separated output better preserves the temporal structure of the aligned reference source after delay and gain compensation.

The correlation trends in Figure 10 follow the SDR ranking closely. The optimized compact FSD retains the highest mean correlation at every SNR, so its advantage is not confined to an energy-ratio metric; it also extends to waveform fidelity after alignment. At 10 dB, for instance, the optimized compact FSD reaches a mean correlation of about 0.303, compared with 0.266 for the frozen reference FSD, 0.267 for PCA-SVD, and 0.173 for AuxIVA. This pattern is consistent with a demixing stage that is better conditioned across frequency and then regularized by a shorter effective time-domain response.

**Figure 11:**
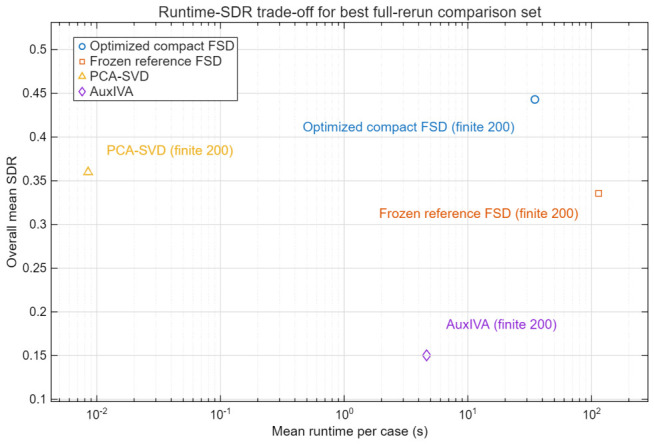
Runtime–SDR trade-off for the selected algorithmic baselines. Each point summarizes one algorithm by its overall mean SDR across 200 matched-source rows and its mean runtime across the 40 trial–SNR cases of the full protocol (5 trials × 8 SNR levels). The horizontal axis is logarithmic in order to visualize the large spread in computational cost.

Figure 11 should be read as a performance–complexity trade-off, not as a one-axis ranking. The optimized compact FSD delivers the highest overall SDR among the selected algorithmic methods, but it is not the fastest algorithm in absolute runtime. The more relevant comparison is structural: the compact parameterization improves on the frozen reference FSD in both accuracy and runtime, while exceeding PCA-SVD and AuxIVA in SDR at the price of added computation. For underwater BSS, that location in the trade-off plane is attractive. The extra iterative demixing cost buys a measurable gain in separation quality, yet the compact configuration remains far less expensive than the frozen reference implementation.

**Figure 12:**
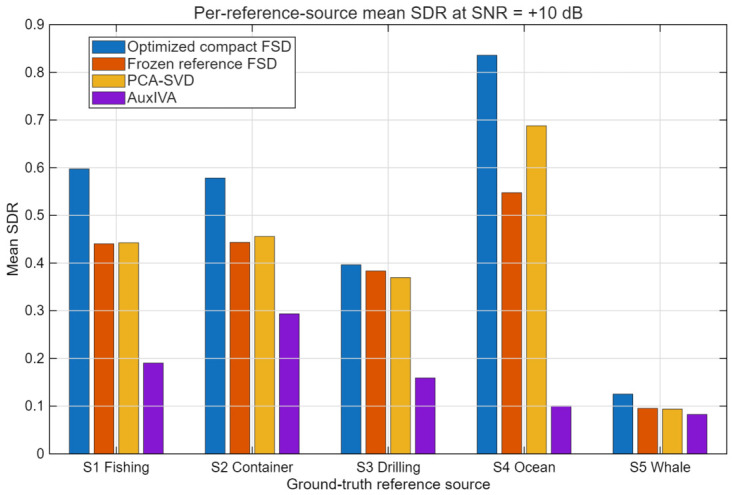
Per-reference-source mean SDR at 10 dB input SNR for the selected algorithmic baselines. The source labels denote the ground-truth reference classes, not the raw order of separated outputs. For each algorithm and reference source class, the bar height is the mean over 5 matched-source metric rows after delay compensation, gain alignment, and reference-source assignment.

Figure 12 resolves the comparison by reference source class at the representative 10 dB operating point. The optimized compact FSD yields the highest mean SDR for all five reference classes in the selected algorithmic group. Reference source 2 denotes the Container Ship class, not the second raw separated output; under the matched-source metric pipeline, its optimized compact FSD mean SDR at 10 dB is approximately 0.578 dB. The margin is especially clear for reference source 4 (Ocean Waves), where the optimized compact FSD reaches approximately 0.836 dB, compared with 0.688 dB for PCA-SVD and 0.548 dB for the frozen reference FSD. Even for reference source 5 (Whale Sounds), which remains difficult for all methods, the optimized compact FSD still preserves the highest mean SDR. That consistency matters in underwater convolutive BSS because it suggests that the gain is distributed across source types rather than being won by favoring one spectral class and degrading another.

**Figure 13:**
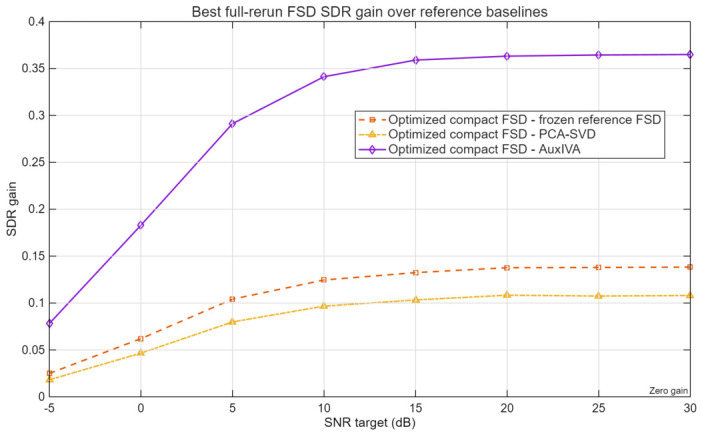
SDR gain of the optimized compact FSD relative to the frozen reference FSD, PCA-SVD, and AuxIVA as a function of input SNR. Each gain value is computed from the corresponding by-SNR mean SDRs, where each mean aggregates 25 matched-source metric rows under the unified ShipsEarBSS protocol. Positive values indicate that the optimized compact FSD achieves higher SDR than the corresponding reference algorithm at the same SNR.

The gain view in Figure 13 makes the same point more directly. All three curves remain positive across the full tested SNR range, so the optimized compact FSD yields higher mean SDR than the frozen reference FSD, PCA-SVD, and AuxIVA at every audited SNR under the shared protocol. The margin is the largest relative to AuxIVA, but it remains positive relative to both the frozen reference FSD and PCA-SVD. Within the selected algorithmic baseline set adopted in this revision, the optimized compact FSD therefore provides the highest observed mean SDR while also improving correlation and reducing runtime relative to the frozen reference FSD.

### 5.4. Ablation Studies

The following ablations isolate three FSD configuration choices under the independent FSD ablation branch: compact demixing-filter support, the number of CPSD blocks, and raw output ordering. The ablation branch uses the same ShipsEarBSS trial/SNR structure and the same matched-source metric implementation as the revised evidence chain, but it is interpreted only as component-level evidence for the tested FSD variants. No scaling-correction ablation is reported here because scaling restoration is not claimed as an independent contribution in this study.

#### 5.4.1. Effect of Compact Demixing-Filter Support

Table 6 reports the sensitivity to the compact support *L* imposed on the demixing filters. In this benchmark, *L* is a regularization parameter for the demixing filters, not the physical length of a BELLHOP channel. The current mixture construction applies BELLHOP arrivals through arrival-based filtering and does not instantiate a fixed-length physical CIR. At 16 kHz, the tested L=64,128,256, and 512 settings correspond to approximately 4, 8, 16, and 32 ms, respectively.

The compact-support variants improve the mean SDR relative to the no-constraint case under the tested protocol. Among the tested settings, L=64 gives the highest mean SDR and the lowest mean MSE, while L=512 behaves close to the no-constraint case. This result supports using compact demixing-filter support as regularization, but it does not imply that L=64 is universally optimal or that *L* covers the complete physical propagation memory.

#### 5.4.2. Effect of the Number of CPSD Blocks

Table 7 reports the sensitivity to *K*, the number of CPSD blocks. Increasing *K* gives finer temporal resolution for nonstationarity, but it also shortens each block and increases the number of matrix operations.

The metric changes across *K* are small compared with the runtime increase. Mean SDR changes by only about 0.002 dB from K=1 to K=10, whereas mean runtime increases from 2.55 s to 46.23 s. Thus *K* is mainly a performance–complexity trade-off in the present protocol. The retained K=5 setting is a practical compromise, not an empirically optimal or generally preferred value.

#### 5.4.3. Effect of Output Ordering

Table 8 compares FSD without algorithmic output ordering against the kurtosis-sorting option. In both cases, evaluation-time reference assignment is used only for metric computation after reconstruction and must not be interpreted as algorithmic permutation correction.

Kurtosis sorting changes the raw algorithmic output order before iSTFT. Under assignment-aware metrics, it does not change SDR, correlation, MSE, or the SIR in the audited ablation; the SIR is 11.604±7.796 for both variants. The appropriate interpretation is therefore limited: kurtosis sorting standardizes raw output order in this implementation, but these results do not show that it solves the frequency-domain permutation ambiguity.

## 6. Conclusions

Separating underwater acoustic sources after convolutive propagation remains difficult because long multipath, spectral overlap, and low SNR undermine the assumptions on which many classical BSS methods rely. This study addressed that problem with FSD, a method centered on multi-block CPSD joint diagonalization in the STFT domain and regularized by a compact time-domain filter constraint. This study also established ShipsEarBSS, a reproducible benchmark that combines real single-source recordings with BELLHOP-derived propagation templates so that underwater convolutive mixtures can be evaluated against known references.

Under the audited repeated-trial protocol, the optimized compact FSD configuration achieved higher mean SDR and waveform correlation than the frozen reference FSD, PCA-SVD, and AuxIVA from −5 to 30 dB within the selected comparison set. The same configuration also reduced runtime relative to the frozen reference FSD. The ablation results further indicate that compact demixing-filter support is useful under the tested protocol, that the effect of *K* is modest relative to its runtime cost, and that kurtosis sorting mainly standardizes raw output order under assignment-aware evaluation. Scaling correction is not claimed as an independent contribution.

The scope of this conclusion should remain explicit. The experiments address the determined case and a simulated benchmark built from controlled deep-water propagation templates rather than sea-trial recordings with unknown source count or rapidly time-varying channels. The present BELLHOP construction uses a 100 Hz arrival file as a traceable multipath delay/amplitude template; it does not constitute a full broadband, frequency-dependent propagation model for the entire 0–8 kHz signal band. Future work should therefore validate the method with multi-frequency or broadband BELLHOP transfer functions, broader propagation conditions, and real sea-trial data. The conclusions about *L*, *K*, and output ordering are empirical under the tested protocol and should not be generalized to all underwater channels. Additional future work should develop stronger cross-frequency permutation alignment and extend the framework to underdetermined settings (M>N), where the second-order FSD framework faces rank-deficient CPSD matrices, the absence of a square demixing matrix, source-count uncertainty, and the need for additional sparsity, clustering, or regularization assumptions.

## Figures and Tables

**Table 1:** Categorization of source signals from the ShipsEar database used in this study.

Category	Description	Files
A	Dredgers, Fishing Vessels, Mussel Boats, Trawlers, Tugboats	1875
B	Motorboats, Pilot Boats, Sailboats	1395
C	Passenger Ferries	3779
D	Ocean Liners, Ro-Ro Vessels	2446
E	Ambient Noise	1140

**Table 2:** Confirmed BELLHOP and ShipsEarBSS benchmark construction parameters.

Item	Confirmed Setting
BELLHOP arrival file	Pos1Azi1freq100Hz.arr
Arrival simulation frequency	100 Hz
Water depth	Approximately 3462 m
Sound-speed profile	Explicit depth-dependent profile in the BELLHOP environment file
Receiver depth in arrival library	10 m
Candidate source depths	6 values spanning 100–1100 m
Candidate ranges	6 values spanning 1–11 km
Source–sensor paths per trial	25 paths for 5 sources and 5 virtual sensors
Trial count and seeds	5 trials, seeds 1001–1005
Source segment format	5 s, 16 kHz, zero-mean and variance-normalized clips
Stored trial artifacts	Reference sources, clean mixtures, channel contributions, arrivals, metadata

**Table 3:** Reference source classes and benchmark roles used in the deep-water BSS benchmark.

Ref. Source ID	Representative Source Type	Benchmark Role
1	Fishing Boat	Structured source to be separated
2	Container Ship	Structured source to be separated
3	Drilling Platform	Structured source to be separated
4	Ocean Waves	Structured source to be separated
5	Whale Sounds	Structured source to be separated

**Table 4:** Traceable methods used in the revised quantitative comparison protocol.

Method	Comparison Role	Methodological Role in the Revised Evaluation
Optimized compact FSD	Proposed configuration	Selected full-rerun FSD configuration from the audited parameter sweep, using $N_{\mathrm{FFT}}=256$ (code parameter T=256), K=5, L=64, max_iter=5000, time-domain truncation, identity initialization, no algorithmic permutation postprocessing, no algorithmic scaling restoration, and channel standardization.
Frozen reference FSD	Formal quantitative baseline	Historical FSD implementation retained as the internal reference for determining whether the optimized compact parameterization yields measurable gains under the same audited protocol.
PCA-SVD	Formal quantitative baseline	Linear subspace reference derived from the leading singular-vector projections of the noisy multichannel mixture, serving as a lightweight non-iterative comparator under the common protocol.
AuxIVA	Formal quantitative baseline	Frequency-domain convolutive BSS reference representing a stronger iterative comparator under the common protocol.

**Table 5:** Paired SDR gain of the optimized compact FSD over selected baselines. Each row uses 200 matched trial–SNR–source pairs. Confidence intervals are 95% paired mean intervals computed from the existing full-rerun TSV files.

Baseline	Mean Gain (dB)	95% CI (dB)	Positive Pairs
Frozen reference FSD	0.108	[0.083, 0.132]	88.5%
PCA-SVD	0.084	[0.066, 0.101]	81.5%
AuxIVA	0.293	[0.251, 0.335]	94.0%

**Table 6:** Filter-length ablation for compact demixing-filter support. Values are mean ± standard deviation over 200 matched-source metric rows (5 trials × 8 SNR levels × 5 reference sources); MSE entries report the mean. Failure count = 0.

Variant	SDR (dB)	Corr.	MSE	Runtime (s)
No constraint	0.336±0.253	0.244±0.116	0.01882	84.78±1.63
L=64	0.435±0.335	0.276±0.130	0.01816	23.62±17.89
L=128	0.405±0.312	0.267±0.125	0.01835	25.74±23.59
L=256	0.390±0.324	0.259±0.128	0.01848	37.75±33.14
L=512	0.336±0.253	0.244±0.116	0.01882	83.76±0.29

**Table 7:** Multi-block CPSD ablation over the number of blocks *K*. Values are mean ± standard deviation over 200 matched-source metric rows; MSE entries report the mean. Failure count = 0.

Variant	SDR (dB)	Corr.	MSE	Runtime (s)
K=1	0.435±0.334	0.276±0.129	0.01816	2.55±3.16
K=3	0.434±0.334	0.275±0.129	0.01817	12.88±10.84
K=5	0.435±0.335	0.276±0.130	0.01816	24.01±18.22
K=8	0.436±0.337	0.276±0.130	0.01815	33.59±29.38
K=10	0.437±0.338	0.276±0.130	0.01814	46.23±36.94

**Table 8:** Permutation-handling ablation. Values are mean ± standard deviation over 200 matched-source metric rows; MSE entries report the mean. Evaluation-time reference assignment is used only for metric computation.

Variant	SDR (dB)	Corr.	MSE	Runtime (s)
No algorithmic ordering	0.435±0.335	0.276±0.130	0.01816	24.11±18.17
Kurtosis sorting	0.435±0.335	0.276±0.130	0.01816	24.14±18.20

## Data Availability

The datasets generated and analyzed in the current study are available upon reasonable request.

## Abbreviations

The following abbreviations are used in this manuscript:

AWGN	Additive White Gaussian Noise
AuxIVA	Auxiliary-Function Independent Vector Analysis
BELLHOP	Beam Tracing for Gaussian Beams by Hamiltonian Optics
BSS	Blind Source Separation
CIR	Channel Impulse Response
CPSD	Cross-Power Spectral Density
DFT	Discrete Fourier Transform
FFT	Fast Fourier Transform
FIR	Finite Impulse Response
FSD	Frequency-Domain Second-Order Decorrelation
HOS	Higher-Order Statistics
ICA	Independent Component Analysis
iSTFT	Inverse Short-Time Fourier Transform
JADE	Joint Approximate Diagonalization of Eigen-matrices
LTI	Linear Time-Invariant
MIMO	Multiple-Input Multiple-Output
MMSE	Minimum Mean Square Error
MVDR	Minimum Variance Distortionless Response
MSE	Mean Squared Error
PCA	Principal Component Analysis
SDR	Signal-to-Distortion Ratio
SIR	Signal-to-Interference Ratio
SNR	Signal-to-Noise Ratio
SOBI	Second-Order Blind Identification
SSP	Sound-Speed Profile
STFT	Short-Time Fourier Transform
SVD	Singular Value Decomposition
ULA	Uniform Linear Array
